# Fahr’s Syndrome in the Setting of Abnormal Calcium-Phosphate Metabolism and Lupus Nephritis

**DOI:** 10.7759/cureus.22298

**Published:** 2022-02-16

**Authors:** Asim Haider, Xiaohui Liang, Muzammil Khan

**Affiliations:** 1 Internal Medicine, BronxCare Health System, Bronx, USA; 2 Internal Medicine, Renaissance School of Medicine at Stony Brook University, Stony Brook, USA; 3 Internal Medicine, Stony Brook University Hospital, Stony Brook, USA

**Keywords:** end-stage renal failure, seizures, hyperthyroidism, fahr’s disease or fahr’s syndrome, lupus nephritis

## Abstract

Fahr’s syndrome is a rare neurodegenerative disorder characterized by bilateral calcifications of the basal ganglia and cerebral cortex. These deposits are made of calcium and phosphorus and are thought to be due to abnormalities in calcium-phosphate homeostasis. The clinical manifestation includes extrapyramidal symptoms (e.g., spastic paralysis), generalized or partial seizures, cognitive impairment, and neuropsychiatric symptoms. Here, we discuss a case of a young female with a medical history of systemic lupus erythematosus (SLE) and lupus nephritis who developed generalized seizures and was found to have extensive bilateral calcifications in the basal ganglia and cerebral cortex. Her laboratory workup showed disorganized calcium-phosphorus homeostasis.

## Introduction

Bilateral calcifications of the basal ganglia and cerebral cortex as seen on computed tomography (CT) or magnetic resonance imaging (MRI) can be suggestive of Fahr’s syndrome, a form of secondary bilateral calcification thought to be caused by a variety of endocrine abnormalities, genetic conditions, infections, or toxins. It has also been described in conjunction with systemic lupus erythematosus (SLE) [1]. The most associated is calcium and phosphorus homeostasis; many case reports describe the relationship between hypoparathyroidism and these bilateral calcifications. This relationship has also been described with hyperparathyroidism, albeit rarer [2]. It is thought that these metabolic conditions lead to abnormal calcium/phosphorous ratio, with precipitation of colloids in cerebral vessels and composition of calcified deposits. These bilateral calcifications are often asymptomatic and discovered on brain imaging.

Beyond the imaging findings of bilateral calcifications, Fahr’s syndrome can also be identified by neuropsychiatric symptoms including cognitive impairment, intellectual disability, extrapyramidal features, psychiatric disorders, motor function deterioration, stroke-like events, spastic paralysis, and generalized or partial seizure [1]. In this article, we discuss the role of hyperparathyroidism and SLE/lupus nephritis in Fahr’s syndrome in this patient with massive cerebral calcifications and its potential neurological manifestations.

## Case presentation

HY was a 34-year-old female with SLE that is complicated by lupus nephritis requiring hemodialysis. In June 2021, while undergoing exchange of her PermaCath, she was noted to have a seizure-like activity that involved brief loss of consciousness, altered mental status (AMS), right arm rigidity, and desaturation to 88% on room air. The loss of consciousness lasted several seconds; by the time the rapid response team arrived, HY was awake, alert, back to baseline, and answering questions appropriately. No postictal symptoms were noted; however, she stated that she had no recollection of the event. At that time, she denied confusion, mental cloudiness, muscle soreness, tongue lacerations, or incontinence. Of note, she reported that she had a similar seizure-like event that led to a fall a few months prior, which was not recorded.

**Figure 1:**
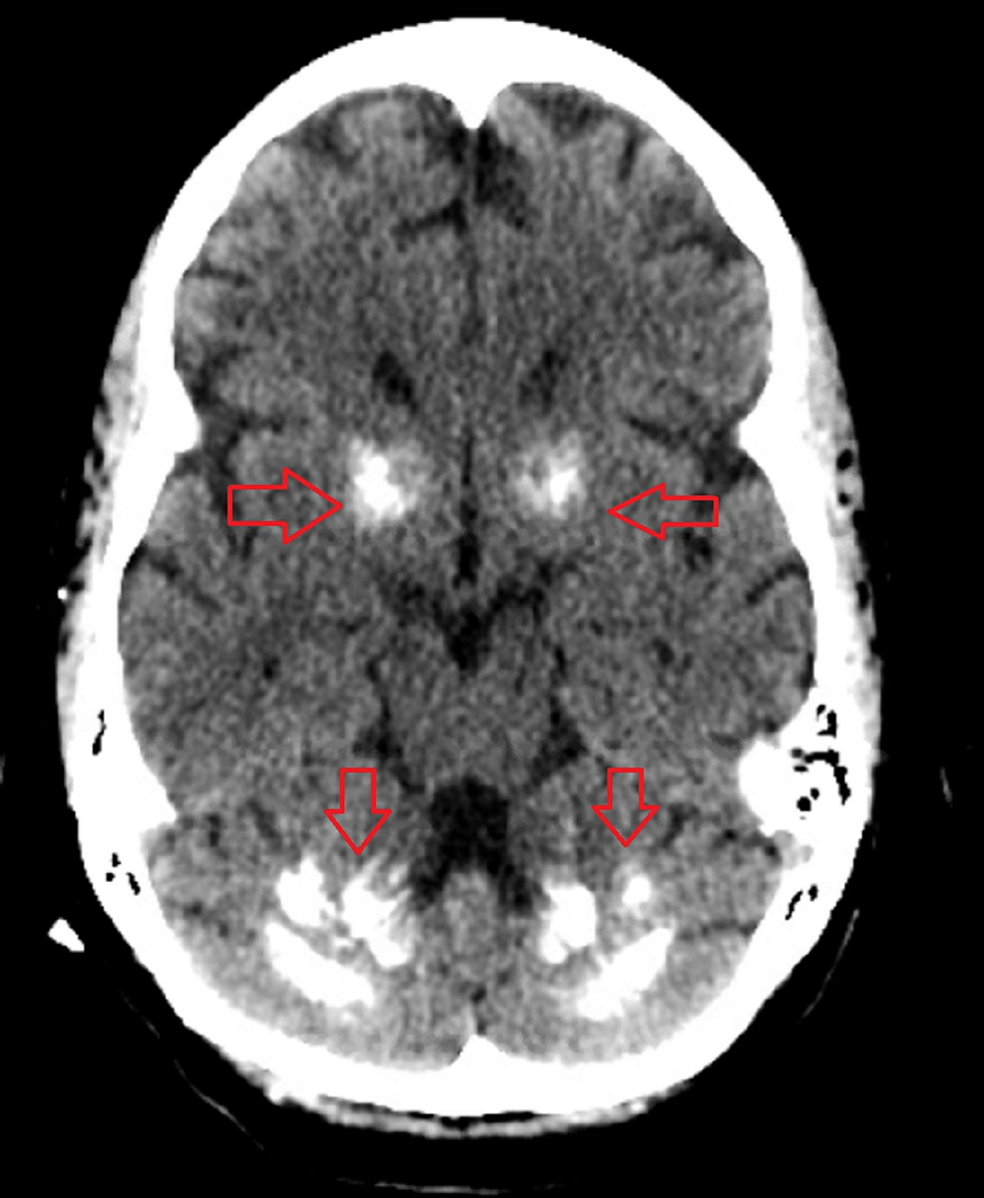
Computed tomography scan of the brain showing bilateral calcification (arrows)

CT imaging of the head without contrast done at the time of rapid response showed no loss of gray-white matter distinction or other sign of acute infarction. However, it demonstrated extensive calcifications involving the bilateral basal ganglia, thalami, medial cerebellar folia, and dentate nuclei bilaterally (Figure 1). Additional linear calcifications were seen within the bilateral cerebral white matter.

An electroencephalogram (EEG) done after the episode did not show any abnormalities. MRI of the brain reinforced susceptibility involving bilateral basal ganglia, bilateral cerebellar hemispheres, and dentate nuclei regions that roughly corresponded with the extensive calcification seen on the CT.

HY has a long history of SLE; she was originally diagnosed at age 15 with arthralgia and skin rash in association with positive ANA titer, Smith antibodies, dsDNA antibodies, and RNP antibodies. She underwent a renal biopsy in 2019 that was consistent with lupus nephritis class V (membranous lupus nephritis.) Unfortunately, she was not consistent with her regimen of lupus medications and diuretics and presented in February 2021 with worsening creatinine and nephrotic range proteinuria for which she was given a few rounds of emergent hemodialysis and after which her kidney function returned.

However, when she was readmitted in April 2021, worsening renal function and oliguria were noted. At that time, blood chemistries revealed a high parathyroid hormone (PTH: 364.5 pg/mL; normal range: 15-65 pg/mL) with an elevated phosphorous level (PO_4_^3-^: 7 mg/dL; normal range: 2.7-4.5 mg/dL) and a normal ionized calcium (Ca^2+^: 4.3 mg/dL; normal range: 4.2-5.2 mg/dL) (Table 1) suggestive of secondary hyperparathyroidism likely due to chronic renal failure.

**Table 1:** Trend of laboratory values (PTH, calcium, and phosphorus) at various points of time PTH: parathyroid hormone

Date	Timepoint	PTH (pg/mL)	Calcium (mg/dL)	Phosphorous (mg/dL)
2/9/2021	First admission	-	7.8	9.8
3/4/2021	First admission midpoint	101.3	8	4.1
3/29/2021	First admission discharge	-	7.3	4.5
4/5/2021	Second admission	364.5	7	7
5/2/2021	Second admission midpoint	70.9	8.1	3.7
6/18/2021	Second admission discharge	-	9.3	4.2

**Figure 2:**
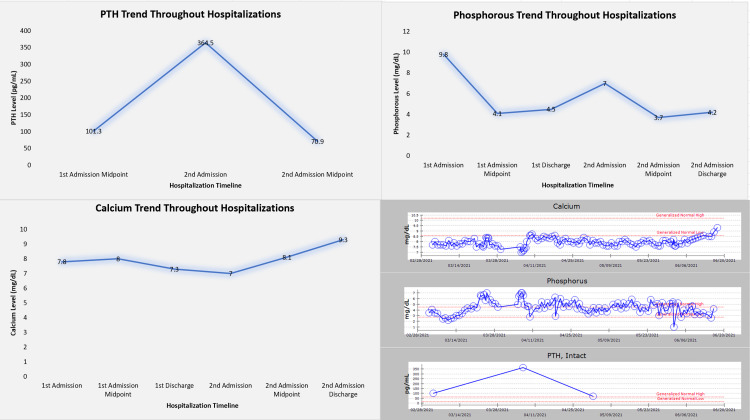
Trend of parathyroid hormone (PTH), calcium, and phosphorus during hospitalization

While these values improved after hemodialysis, a review of her blood chemistries since she first presented in February with her original lupus nephritis flare suggests that she had many months of disordered calcium-phosphorus metabolism (Figure 2).

No other underlying cause of HY’s bilateral calcifications and seizure-like episode could be found. Her immunology studies do not support the diagnosis of cerebral lupus; her cardiolipin IgA, IgG, and IgM antibodies were within reference ranges, and her serum C3 and C4 levels were within normal limits.

HY originally did not have any observed major neurological or psychological abnormalities that would suggest an underlying Fahr’s syndrome until her seizure-like episode as mentioned earlier. On psychiatric evaluation during admission, HY was diagnosed with adjustment disorder with depressed mood, likely due to her frequent and extended hospital stays; she endorsed low mood, anhedonia, difficulty sleeping, difficulty eating, and loss of hope around her condition. Otherwise, she has no history of depression or alcohol or drug abuse. She also has no family history of psychiatric illness, neurological illness, dementia, or Fahr’s disease. Furthermore, her siblings were healthy. Genetic testing was not offered to our patient because of a lack of resources.

## Discussion

Fahr’s syndrome is often first diagnosed when CT imaging reveals bilateral intracerebral calcifications in the cerebral gray matter such as those seen in HY. However, the clinical presentations and age at the time of presentation vary greatly [3]. While basal ganglia calcifications are common idiopathic findings in older patients, it is more concerning in our patient given her younger age. Those incidental calcifications are also usually asymptomatic; thus, when our patient exhibited loss of consciousness and upper extremity rigidity, one of the main clinical manifestations of Fahr’s syndrome, there was more suspicion of a disease process [4].

Fahr’s syndrome is associated with a variety of clinical features including neurological symptoms, movement disorders, and neuropsychiatric symptoms [5]. Reportedly, between 33% and 50% of those with Fahr’s syndrome have neurological symptoms, which include loss of consciousness, convulsions, and spasticity [6]. These symptoms were seen in HY and, in conjunction with her brain imagining findings and history, are highly suggestive of Fahr’s syndrome. Although her EEG did not review any abnormalities, this study is limited by timing. Furthermore, seizures are rarely reported as the presenting symptoms of Fahr’s syndrome [3].

The pathology of these calcifications and symptoms are thought to be related to endocrine disorders, namely, hypoparathyroidism or pseudohypoparathyroidism [7,8]. It has also been reported in association with hyperparathyroidism [2]. When incorporating the findings in this case, the gray matter calcifications appear to be a result of abnormal calcium/phosphate ratios regardless of the underlying cause. The dysregulation caused by HY’s chronic renal failure due to lupus nephritis may have contributed to her calcifications and symptoms. Given the extended time period of poor medication adherence and uncontrolled lupus nephritis flare, it is likely that these calcifications insidiously grew until she became symptomatic.

Interestingly, Fahr’s syndrome has also been reported in conjunction with SLE itself [1]. The case presented involved an older patient with a similar length of disease. However, it appears that her disease was more well-controlled and did not have any major neurological abnormality, largely complaining of insomnia and mild depression. Given this association, it is possible that our patient presented at an earlier age and with more major neurological abnormalities due to poor disease control.

The prognosis of Fahr’s syndrome is difficult to predict. Studies have not shown any correlation between the extent of calcification and the severity of disease [9]. A long-term follow-up and detailed family history are required to confirm the diagnosis. There is no proven treatment for this disease. Most of the patients are managed via symptomatic support [10].

## Conclusions

Our study strengthens the association between disordered calcium-phosphorous metabolism and Fahr’s syndrome. The disordered metabolism mentioned in the literature thus far primarily involves hypoparathyroidism and primary hyperparathyroidism. However, this, to the best of our knowledge, is the first reported case of massive cerebral calcifications in a patient with SLE complicated by lupus nephritis. Fahr’s syndrome has a varied clinical presentation that includes the AMS and rigidity experience seen in this case. However, the common bilateral cranial calcifications seen in these patients can be due to general calcium-phosphate metabolism impairment, regardless of its origin.
